# Intelligent Sensing, Control and Optimization of Networks

**DOI:** 10.3390/s24051648

**Published:** 2024-03-03

**Authors:** Guanghui Wen, Junjie Fu, Jialing Zhou

**Affiliations:** 1 Department of Systems Science, School of Mathematics, Southeast University, Nanjing 211189, China; fujunjie@seu.edu.cn; 2 Advanced Research Institute of Multidisciplinary Sciences, Beijing Institute of Technology, Beijing 100081, China; jlzhou@bit.edu.cn

The development of many modern critical infrastructures calls for the integration of advanced technologies and algorithms to enhance the performance, efficiency, and reliability of network systems [1,2]. These technologies and algorithms enable networks to sense their environment, make intelligent decisions, and optimize their operations in real-time, based on the collected data and information [3,4,5]. The application areas include smart cities, smart homes, industrial automation, transportation systems and so on [6,7,8]. As nowadays cyber-physical networks become more complex and interconnected, the demand for high-performance, reliable, and secure networks continues to grow which has also received much attention from various scientific fields [9,10].

The present Special Issue mainly focuses on future complex cyber-physical networks (CCPNs) and smart sensors. To fully release the potential of these networked systems, effective control and optimization strategies are needed to enable collaboration of the interconnected components. Furthermore, data-based technologies have also seen increasing usage in the sensing, control and optimization process of the various types of networks. The development of these technologies will significantly enhance the stability, reliability and efficiency of future CCPNs. In view of the above need, a call for papers of this Special Issue was carefully prepared by the guest editors and after posting it online, widespread attention from various areas ranging from mathematics, computer science, automation science, artificial intelligence was drawn. More than 30 submissions were received, in which the topics covered different aspects of sensing, control and optimization of networked systems. All the submitted manuscripts have went through a thorough peer-refereeing process. Based on the reviewers’ evaluations and the Guest Editors’ comments, 10 original research articles and 1 review article were finally accepted, and the contents of the accepted papers are briefly summarized below.

The article by Nan et al. (contribution 1) considers the problem of precisely recognizing the feature information of remote sensing images. The main challenges are to handle the different depths and the non-uniform feature distributions. The disadvantages of the traditional Unet are analyzed and new methods are proposed to achieve optimal performance. The obtained results have the potential to aid the automatic planning of power transmission lines.

The article by Yu et al. (contribution 2) proposes a novel optimal distributed finite-time fusion filtering method. Compared with existing results, the dynamic communication weights are used to achieve faster convergence speed. A new concept of limited iterations of global information aggregation is proposed and matrix weight fusion is employed to achieve optimal estimation.

The article by Nan et al. (contribution 3) introduces an improved Dijkstra algorithm based on adaptive resolution grid to assist manual transmission line planning. The redundant grids are firstly reduced by a new segmenting method. Then, an improved Dijkstra algorithm is introduced to find the lowest-cost transmission line. The proposed method was shown to achieve higher planning efficiency and faster speed.

The article by Shang et al. (contribution 4) proposes a matrix weight fusion method with a feedback structure to achieve optimal filtering for sensor networks with correlated noises and packet dropout. A predictor with a feedback structure is the key step to reduce the covariance of the fusing results.

The article by Qin et al. (contribution 5) focuses on the traversal exploration and path coverage problem of target regions using multiple agents. The rapidly exploring random trees (RRT) algorithm is modified in two ways. First, geodesic distance is used for the corner case of narrow regions. Second, the Dijkstra algorithm is used to reduce the number of turns in the path. The proposed method has multiple advantages such as complete coverage, wide application to area shapes and minimum average waiting time.

The article by Sun et al. (contribution 6) considers the map-merging problem for UAVs to improve the efficiency of distributed exploration. The main difficulties include the absence of a common reference coordinate system and the missing of relative position information of UAVs. A new algorithm is introduced which considers the dissimilarity between the overlapping regions. The improved genetic algorithm is proposed, which has a stronger global search capability.

The article by Sun et al. (contribution 7) studies the area coverage control problem based on the time cost metric for a robot network. The time-varying density function is considered to handle movable objects in the task area. A two-phase strategy was employed to realize robust coverage control, which minimizes the time cost metric in a fixed time.

The article by Bao et al. (contribution 8) proposes a novel adaptive deskewing algorithm for document images to increase the efficiency of optical character recognition. The proposed algorithm contains several innovative steps which successfully increase the accuracy for information analysis.

The article by Liu et al. (contribution 9) proposes an intelligent positioning method for a class of climbing robot which can be applied to transmission towers. The proposed strategy combines the three-dimensional location model of transmission tower and the visual sensor data from the sensors. The results help the robots to adjust to the working area and improves the working accuracy during the climbing.

The article by Zhu et al. (contribution 10) aims at handling the nonlinearity and inaccuracy of the model for pneumatic control of the valve position. A new method based on a fractional-order PID controller is proposed, which employs an optimization algorithm to tune the parameters. The proposed strategy combines model identification and advanced control and achieves better performance than a traditional PID controller.

The review article by Wan et al. (contribution 11) focuses on some recent developments and methodologies for the secure control of complex cyber–physical networks. Both the single type of cyberattack and hybrid cyberattacks are surveyed. The secure control aspects are also considered, which include strategies from both topology and control perspectives.

We would like to thank the authors for their contributions and acknowledge all the reviewers for their time and effort in assessing the manuscripts. The gratitude is also extended to the Editor-in-Chief and the Editorial Office of *Sensors* for their kind support.
